# Longitudinal Model Shifts of Machine Learning–Based Clinical Risk Prediction Models: Evaluation Study of Multiple Use Cases Across Different Hospitals

**DOI:** 10.2196/51409

**Published:** 2024-12-13

**Authors:** Patricia Cabanillas Silva, Hong Sun, Mohamed Rezk, Diana M Roccaro-Waldmeyer, Janis Fliegenschmidt, Nikolai Hulde, Vera von Dossow, Laurent Meesseman, Kristof Depraetere, Joerg Stieg, Ralph Szymanowsky, Fried-Michael Dahlweid

**Affiliations:** 1 Dedalus HealthCare Antwerp Belgium; 2 Provincial Key Laboratory of Multimodal Perceiving and Intelligent Systems Jiaxing University Jiaxing China; 3 Engineering Research Center of Intelligent Human Health Situation Awareness of Zhejiang Province Jiaxing University Jiaxing China; 4 Institute of Anaesthesiology and Pain Therapy Heart and Diabetes Centre North Rhine Westphalia University Hospital of Ruhr-University Bochum Bad Oeynhausen Germany

**Keywords:** model shift, model monitoring, prediction models, acute kidney injury, AKI, sepsis, delirium, decision curve analysis, DCA

## Abstract

**Background:**

In recent years, machine learning (ML)–based models have been widely used in clinical domains to predict clinical risk events. However, in production, the performances of such models heavily rely on changes in the system and data. The dynamic nature of the system environment, characterized by continuous changes, has significant implications for prediction models, leading to performance degradation and reduced clinical efficacy. Thus, monitoring model shifts and evaluating their impact on prediction models are of utmost importance.

**Objective:**

This study aimed to assess the impact of a model shift on ML-based prediction models by evaluating 3 different use cases—delirium, sepsis, and acute kidney injury (AKI)—from 2 hospitals (M and H) with different patient populations and investigate potential model deterioration during the COVID-19 pandemic period.

**Methods:**

We trained prediction models using retrospective data from earlier years and examined the presence of a model shift using data from more recent years. We used the area under the receiver operating characteristic curve (AUROC) to evaluate model performance and analyzed the calibration curves over time. We also assessed the influence on clinical decisions by evaluating the alert rate, the rates of over- and underdiagnosis, and the decision curve.

**Results:**

The 2 data sets used in this study contained 189,775 and 180,976 medical cases for hospitals M and H, respectively. Statistical analyses (*Z* test) revealed no significant difference (*P*>.05) between the AUROCs from the different years for all use cases and hospitals. For example, in hospital M, AKI did not show a significant difference between 2020 (AUROC=0.898) and 2021 (AUROC=0.907, Z=–1.171, *P*=.242). Similar results were observed in both hospitals and for all use cases (sepsis and delirium) when comparing all the different years. However, when evaluating the calibration curves at the 2 hospitals, model shifts were observed for the delirium and sepsis use cases but not for AKI. Additionally, to investigate the clinical utility of our models, we performed decision curve analysis (DCA) and compared the results across the different years. A pairwise nonparametric statistical comparison showed no differences in the net benefit at the probability thresholds of interest (*P*>.05). The comprehensive evaluations performed in this study ensured robust model performance of all the investigated models across the years. Moreover, neither performance deteriorations nor alert surges were observed during the COVID-19 pandemic period.

**Conclusions:**

Clinical risk prediction models were affected by the dynamic and continuous evolution of clinical practices and workflows. The performance of the models evaluated in this study appeared stable when assessed using AUROCs, showing no significant variations over the years. Additional model shift investigations suggested that a calibration shift was present for certain use cases (delirium and sepsis). However, these changes did not have any impact on the clinical utility of the models based on DCA. Consequently, it is crucial to closely monitor data changes and detect possible model shifts, along with their potential influence on clinical decision-making.

## Introduction

In recent years, machine learning (ML) algorithms for clinical risk predictions have emerged as a promising tool and have been widely used in health care applications [1-5]. However, most of such applications have been developed and evaluated on retrospective data [6,7], and their performance in live clinical settings remains understudied.

Wu et al [8] highlighted the importance of evaluating artificial intelligence (AI)–based medical devices with live clinical settings over different sites to address the shortcomings, such as overfitting to training data and bias against underrepresented subgroups. The evaluation of models’ performances in live clinical settings is drawing more attention and is becoming more adopted in the field [9,10]. In our previous paper, we investigated clinical risk prediction models for heterogeneous patient populations from different hospitals and across different clinical use cases: delirium, sepsis, and acute kidney injury (AKI). By evaluating these models in both retrospective [7] and live clinical settings [11], we demonstrated that the diversity in patient populations translates into improved generalizability and a more comprehensive understanding [11]. A cohort study was also carried out to evaluate the clinical usefulness of our delirium prediction model [12]. These results led to a later review to conclude that our approach demonstrates the best clinical utility and highest performance when evaluated beyond the development setting [13].

However, even when evaluated in clinical settings, there remains a critical challenge that poses a significant threat to the reliability and performance of these models, which is the phenomenon of model shift [14-16]. Most ML-based prediction models use ML algorithms that leverage statistical methods to learn from clinical data. When clinical prediction models are deployed in nonstationary clinical environments [17], model deteriorations are observed over time in response to the dynamic nature of clinical environments [18]. The effectiveness and generalizability of ML-based clinical risk prediction models are greatly impacted when in such situations. As an example, during the COVID-19 pandemic, the alert rate of a sepsis prediction model was reported as more than double compared to that preceding the COVID-19 pandemic. As a consequence, the hospital had to shut down the prediction service [19,20].

This study intended to evaluate the model shift in ML-based clinical risk prediction models. Following the work of our previous study [7,11], we aimed to generalize our findings by applying the investigation to the delirium, sepsis, and AKI use cases at 1 community hospital and 1 specialized hospital. In this study, we used retrospective data to train different prediction models on clinical data from earlier years and then evaluated the model shift, as well as its impact on clinical decision-making, on data from later years, thus mimicking the situation of deploying prediction models in the real world. The evaluation covered periods preceding and during the COVID-19 pandemic, which allowed us to evaluate the impact of COVID-19. We investigated the impact of a model shift on model performance, model calibration, and clinical decisions. To the best of our knowledge, this is the first report that evaluated the impact of a model shift on such a scale.

## Methods

### Ethical Considerations

In accordance with Article 15.1 of the Professional Code for Healthcare Professionals of Germany, ethical approval is only required if human body materials are used or if the used data can be traced to a particular individual [21]. As no personal data were collected for this project and all used data were anonymized in compliance with the ethical and data protection obligations established by the European Commission [22], consultation or evaluation by an ethics committee was not required.

### Study Design

We used a scalable method development approach to generate clinical risk prediction models for various conditions at 2 German hospitals, the Medius Klinik Nürtingen and the Herz- und Diabeteszentrum Nordrhein-Westfalen Bad Oeynhausen, referred to as hospitals M and H, respectively. Our previous investigations showed that our models’ performance from live clinical workflows did not deteriorate compared to retrospective data. For instance, when comparing the area under the receiver operating characteristic curve (AUROC) on average across the 3 use cases and hospitals from live clinical workflows to that from retrospective data, the AUROC decreased slightly by 0.6 percentage points (from 94.8% to 94.2% at discharge) [7,11]. This study incorporated patient data from 2009 to 2021, which included demographic information, such as age and sex; clinical information, such as laboratory results; clinical notes, such as admission letters or nursing notes; vital signs; historical records; and medication. Tables S1 and S2 in Multimedia Appendix 1 provide an overview of data characteristics over the years for the 2 hospitals, respectively. We focused on 3 clinical use cases: delirium, sepsis, and AKI. Table 1 displays the incidence of each condition at each hospital.

**Table 1:** Incidence for each use case in both hospitals.

Hospital	AKI^a^ incidence (%)	Delirium incidence (%)	Sepsis incidence (%)
M	16.41	2.22	1.50
H	23.35	1.15	2.13

^a^AKI: acute kidney injury.

### Analysis and Evaluation

**Figure 1:**
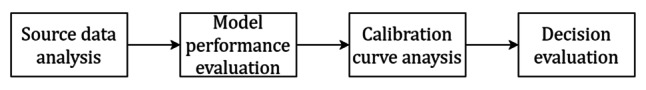
Overview of the investigation methodology.

We performed analyses and evaluations from different perspectives to systematically assess the model shift and its impact. Figure 1 provides an overview of these steps: First, we conducted a comprehensive analysis of the source data to detect any changes in them over time. Second, we evaluated the performance of the prediction model to determine whether there was any deterioration. Third, we examined any potential shift in the calibration curves. Lastly, we performed a decision analysis to determine whether there was a decline in clinical decision-making.

### Data Source

As clinical prediction models may be negatively impacted by changes in clinical settings over time [18], one of the first steps in avoiding a model shift is to analyze the source data and look for changes in them. First, we looked at the number of samples per year, as this could drastically change. Second, it was important to track the incidence of the condition over the years as it could have been altered due to modifications in the coding system, the hospital protocol, or the appearance of new risk factors that cause an increase in incidence—for example, SARS-Cov2 as a risk factor for sepsis [23]. Third, it was necessary to closely examine data set characteristics. Among the key points were gender distribution over time, the availability of features throughout the years, and the length of observations and patient stay over the years.

### Model Development

The prediction model design, data preparation, and training were implemented in an automated pipeline that provided a standardized method to install, configure, and execute the data preparation process, model training, and evaluation on a deployment-specific system. The automated pipeline started with source data analysis to detect abnormalities in the source data. It also automatically adapted the hyperparameters for the model training process. Risk prediction models were then generated based on the hospital data with such an automated pipeline and, therefore, calibrated with the electronic health record (EHR) data of the hospital. The generated models were automatically evaluated, and acceptance criteria were verified throughout the evaluation procedure.

Before data preparation, we applied the following inclusion criteria: (1) patients should be 18 years or older and must have a birth year available; (2) medical cases should contain information about the gender, the department, and the reason for admission; and (3) stay in the hospital should be for a maximum of 90 days. Next, input features were generated using a common data preparation pipeline, which used all the available data generated during the patient’s stay. The features came from structured data, such as laboratory results, vital signs, and historical records, along with unstructured data, such as clinical notes. Details about the number of features used by the model for both hospitals are provided in Table S3 in Multimedia Appendix 1.

For preparing unstructured features, we developed a natural language processing (NLP) pipeline in 2 steps. The initial step involved using a trained TinyBERT model to perform named entity recognition (NER) on clinical text data, identifying disorders and clinical findings. The extracted entities were then passed as an input to a named entity normalization (NEN) model [24]. The NEN model maps the extracted entities to the *German Modification of the International Statistical Classification of Diseases and Related Health Problems, Tenth Revision* (ICD-10-GM) codes. This 2-step process enhances the precision of information extraction and ensures a consistent representation of clinical concepts. (For more details, see the *NLP Models* section in Multimedia Appendix 2). The labels for sepsis and delirium were assigned according to the ICD-10-GM codes at discharge. The AKI labels were assigned based on the Kidney Disease Improving Global Outcomes (KDIGO) criteria [25].

To ensure the reliability of the study, the data sets were strictly split into training and evaluation sets at the patient level. Additionally, approximately 10% of the training set was exclusively reserved for validation, calibration, and threshold selection. The training set for hospital H included data from 2017 to 2018, while that for hospital M comprised data from 2009 to 2017. Consequently, the evaluation set contained the most recent years, spanning from 2019 for hospital H and from 2018 for hospital M. The choice of these specific years was due to differences in incidence across the years because of mislabeling (for more details, see data source analysis in the *Results* section).

All the available information belonging to the same hospital stay was first aggregated to obtain a complete training record. Additionally, to mitigate the situation where the model was requested to generate predictions with limited information available, we applied data augmentation in the training set to generate a partial record. The partial data were constructed based on subsets of features of the original record and, combined with the complete record, enhanced the robustness of the clinical risk prediction model. Nevertheless, it is important to note that this approach was applied only to the training data, while the test set remained unmodified to reflect the real-life distribution of the population. The impact of this data augmentation was evaluated in our previous work [7], where the performance 24 hours/1 day/the next day after admission showed an improvement of around 4% in the AUROC. Furthermore, to avoid potential biases due to high class imbalance, the controls were downsampled to a 1:5 case:control ratio before training. More detailed information about the model development approach can be found in our previous paper [7].

Next, a clinical risk prediction model was trained separately for each condition (sepsis, delirium, and AKI) and each hospital (M and H), applying a standard model training methodology using/on transformer models [26]. As a result, we obtained 6 independent models. For the model training process, the previously mentioned features were used as inputs and the labels as targets of the model. However, it is important to note that certain features exhibited unreasonably strong correlations with the condition. Consequently, these features, identified as leaking features, were removed from the training data. Roughly, 2 categories of leaking features were considered: reverse causal correlations, which correspond to features that typically occur after onset (or clinical suspicion), and strong cofounders, such as the presence of specific but unrelated lab tests only taken in a high-risk population. The list of these features, defined by in-house clinical professionals, can be found in Table S4 in Multimedia Appendix 1.

### Model Performance

A common pitfall of clinical prediction models is that their performance can deteriorate over time. Therefore, a key aspect to explore is the ability of the model to discriminate and sustain its performance over time. There are different metrics that can be used (eg, accuracy, sensitivity, specificity, precision) to evaluate a model’s performance. In this study, we applied 2 methods to investigate the performance of a model: first, the AUROC, as it is an assessment metric that remains unaffected by incidence or threshold selection, and second, the calibration shift, which is the disagreement between the predicted probability and the observed number of events.

To detect a calibration shift, a well-calibrated model that makes probabilistic predictions that match real-world probabilities is required. There are several methodologies for calibrating a model, such as Platt scaling [27], isotonic regression [28], and temperature scaling (which is a single-parameter variant of Platt scaling) [29], among others. In this study, we used Platt scaling, which is more suitable for small data sets (like our sepsis and delirium data sets). Additionally, for plotting the calibration graph, we used a quantile binning approach, where each bin has the same number of samples, which reduces the noise in the curve by preventing different bins from having an unbalanced number of samples. There is no 1 unique way to measure the calibration of predictive models. Although some methods are frequently used and have specific strengths, all have limitations [30]. In this analysis, we focused on 2 metrics: expected calibration error (ECE) and maximum calibration error (MCE). The ECE measures the weighted mean absolute difference between confidence and accuracy, while the MCE measures the maximum difference between average confidence and accuracy across bins. In both metrics, larger values indicate greater miscalibration.

We generated calibration curves for each year within the test set , which allowed us to more easily detect a calibration shift by identifying deviations between the different curves. We observed calibration shifts for sepsis and delirium but not for AKI. Consequently, we expanded our experiments to assess whether the shift was related to the intrinsic nature of different conditions and also whether it was related to other factors, such as data size or incidence. Although other factors can contribute to the presence or absence of a shift, we investigated how the former 2 impacted the results in AKI, as no evidence of a shift was observed for this condition. Furthermore, the AKI use case exhibited a larger sample size and a higher incidence rate compared to the other 2 use cases. This provided us with an opportunity to conduct the following experiments: First, we randomly downsampled the AKI data set to assess the shift on different data scales. Second, we artificially reduced the incidence rate of AKI in the testing and calibration data sets to match the delirium incidence rate. These experiments aimed to assess the influence of both data size and incidence rate on the outcomes and whether the observed shift in sepsis and delirium is a consequence of a lower data size or incidence.

### Decision Evaluation

The system calculated predictions throughout the hospitalization, and when a threshold was reached, an alert was shown. An alert may appear as soon as the patient is registered in the system if their past medical history is predictive enough. We conceptually created 5 categories (instead of the typical 4):

- True negative (TN; control): an alert is not shown correctly.
- False positive (FP; control): an alert is incorrectly shown at some point during the stay.
- False negative (FN; case): an alert is incorrectly not shown at all, although the patient has developed the condition at some point during the stay.
- True positive (TP; case): an alert is correctly shown before onset.
- Arguably FN/TP (case): an alert is correctly shown but not strictly before onset. The risk prediction is then diagnostic rather than strictly predictive, similar to other risk scores, such as the Confusion Assessment Method for the Intensive Care Unit (CAM-ICU) or Delirium Observation Scaling (DOS).

A severe limitation of ICD codes is that they convey no information about the time of onset of a condition. We were, therefore, unable to distinguish between the last 2 groups, so we evaluated the occurrence of an alert at any time from admission until discharge (as opposed to no alert at all) and compared this alert rate to the finally assigned diagnosis.

Model performance evaluation normally focuses on discrimination metrics; however, it does not assess whether the risk model enhances clinical decision-making. Accordingly, we undertook an investigation to determine the potential impact on decision-making over time by analyzing alert rates and over- and underdiagnosis rates and performing decision curve analysis (DCA). First, we investigated whether there was any increase or decrease in alert rates across the years. Second, we examined the changes in over- and underdiagnosis rates. Overdiagnosis refers to overtreatment without any discernible benefit to the patient, which typically occurs in sensitive models, and is defined as the false-positive rate (FPR). Underdiagnosis, in contrast, refers to a failure to correctly diagnose a condition and is defined as the false-negative rate (FNR) [31,32]. The FPR and FNR are affected by threshold selection. Thus, in the pursuit of achieving an equilibrium between these 2 metrics, our threshold selection approach consisted of selecting a threshold that minimized the difference between under- and overdiagnosis rates. This threshold selection was performed in an independent set (10% of the training set) and after calibrating the model. Lastly, we performed DCA, where we assessed whether the benefit of using our model to make a clinical decision decreased over time. DCA allows us to evaluate and compare the clinical utility of predictive models using nonparametric statistical methods [33,34]. This method illustrates the net benefit related to a specific decision strategy across a spectrum of probabilities. Its application ensures that clinical decisions are not solely reliant on discriminative metrics but also consider the potential harms and benefits associated with such decisions. The net benefit is calculated according to the following formula:



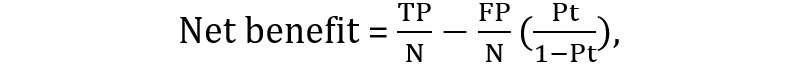



where N is the total number of predictions and is the threshold probability.

## Results

### Data Source Analysis

The data sets used in this study contained 189,775 and 180,976 medical cases for hospitals M and H, respectively. The distribution and characteristics of the data sets for both hospitals are shown in Tables S1 and S2 in Multimedia Appendix 1. Hospital M is a community hospital, while hospital H is a specialized center for diabetes and heart disease, with a high volume of cardiac surgery and invasive, transcatheter procedures and intensive care. The 2 data sets exhibit common characteristics with respect to an increasing trend in the length of stay, resulting in a greater number of features and longer observations, and consistency in the proportion of women and men throughout the years (Tables S1 and S2 in Multimedia Appendix 1). However, it is important to note that there are some differences between the 2 data sets or different use cases. For instance, medication was not available as a feature until 2016 in hospital M and until 2018 in hospital H. Moreover, the incidence of sepsis and delirium in earlier years was lower than in later years, with a more notable disparity observed in delirium at hospital H. The increase in the delirium incidence rate is coincident with extensive efforts to improve the recognition and treatment of delirium. Based on these findings, we decided to use only data from the most recent years, specifically from 2017 for hospital H. This decision was made to mitigate the potential influence of mislabeling arising from the data source. Medical cases whose admission date was earlier than 2017 were removed. The real-life incidence for the validation and evaluation test was therefore not affected. As aforementioned, the case:control balance for the training data set was set to 1:5.

### Model Performance

The performance of the clinical risk prediction model was evaluated at the end of the patient’s hospital stay due to the lack of the time of onset and to avoid inconsistencies when the predictions were evaluated.

#### Performance Evaluation

Model evaluation results are presented in Tables 2 and 3 by showing AUROC and area under the precision recall curve (AUPRC) values. The AUPRC is a relevant metric when dealing with imbalanced data and detecting positive samples are of greater interest and concern. However, as the AUPRC is highly influenced by incidence, the model was evaluated based on the AUROC, which, in contrast to other metrics, such as sensitivity, specificity, precision, and the AUPRC, is independent of the threshold and incidence. Thus, it allowed us to compare model performance across different years. Table 2 shows that AUROC values remained stable for the 2 use cases at hospital M, with mean 90.37% (SD 0.32%), mean 96.95% (SD 0.50%), and mean 95.92% (SD 0.41%) for AKI, delirium, and sepsis, respectively. Similarly, Table 3 presents the values for hospital H, and the values for AKI, delirium, and sepsis were mean 91.25% (SD 0.29%), mean 94.24% (SD 0.44%), and mean 97.95% (SD 0.41%), respectively.

**Table 2:** Model performance and incidence in the test set of hospital M.

Use case and metrics		Years			
		2018	2019	2020	2021
**AKI^a^**					
	Incidence (%)	14.57	14.81	14.95	17.64
	Alert rate (%)	22.36	22.62	23.48	26.56
	AUROC^b^ (%)	90.54	90.40	89.84	90.69
	AUPRC^c^ (%)	65.48	65.87	64.99	71.01
**Delirium**					
	Incidence (%)	2.18	1.92	3.17	2.63
	Alert rate (%)	10.75	11.61	13.44	15.37
	AUROC (%)	97.55	96.86	97.21	96.18
	AUPRC (%)	42.93	34.94	51.26	40.89
**Sepsis**					
	Incidence (%)	1.67	1.49	1.24	1.66
	Alert rate (%)	11.86	13.12	14.65	17.17
	AUROC (%)	96.29	96.37	95.45	95.55
	AUPRC (%)	40.98	32.85	34.14	39.81

^a^AKI: acute kidney injury.

^b^AUROC: area under the receiver operating characteristic curve.

^c^AUPRC: area under the precision recall curve.

**Table 3:** Model performance and incidence in the test set of hospital H.

Use case and metrics		Years		
		2019	2020	2021
**AKI^a^**				
	Incidence (%)	25.22	25.98	25.58
	Alert rate (%)	32.70	34.36	32.76
	AUROC^b^ (%)	90.83	91.51	91.40
	AUPRC^c^ (%)	80.58	82.59	81.71
**Delirium**				
	Incidence (%)	3.25	3.26	2.86
	Alert rate (%)	20.56	21.25	20.46
	AUROC (%)	94.70	94.39	93.64
	AUPRC (%)	37.32	34.43	30.91
**Sepsis**				
	Incidence (%)	3.70	3.86	3.89
	Alert rate (%)	12.82	12.47	12.79
	AUROC (%)	97.37	98.12	98.35
	AUPRC (%)	73.63	77.04	77.78

^a^AKI: acute kidney injury.

^b^AUROC: area under the receiver operating characteristic curve.

^c^AUPRC: area under the precision recall curve.

These findings indicate low variations in the performance metrics; moreover, statistical tests were conducted to discern whether the variations in performance metrics over the years were statistically significant. First, we computed the SEs of the AUROCs using Hanley and McNeil’s method [35]. Next, we performed a *Z* test to assess whether there were significant differences between different AUROCs. We did not observe any significant differences between the AUROCs across the different years for all use cases and all hospitals (*P*>.05). Detailed results are presented in Tables S5 and S6 in Multimedia Appendix 1. These results demonstrate that the model’s performance did not exhibit persistent deterioration over time at either hospital.

#### Calibration Curve Analysis

**Figure 2:**
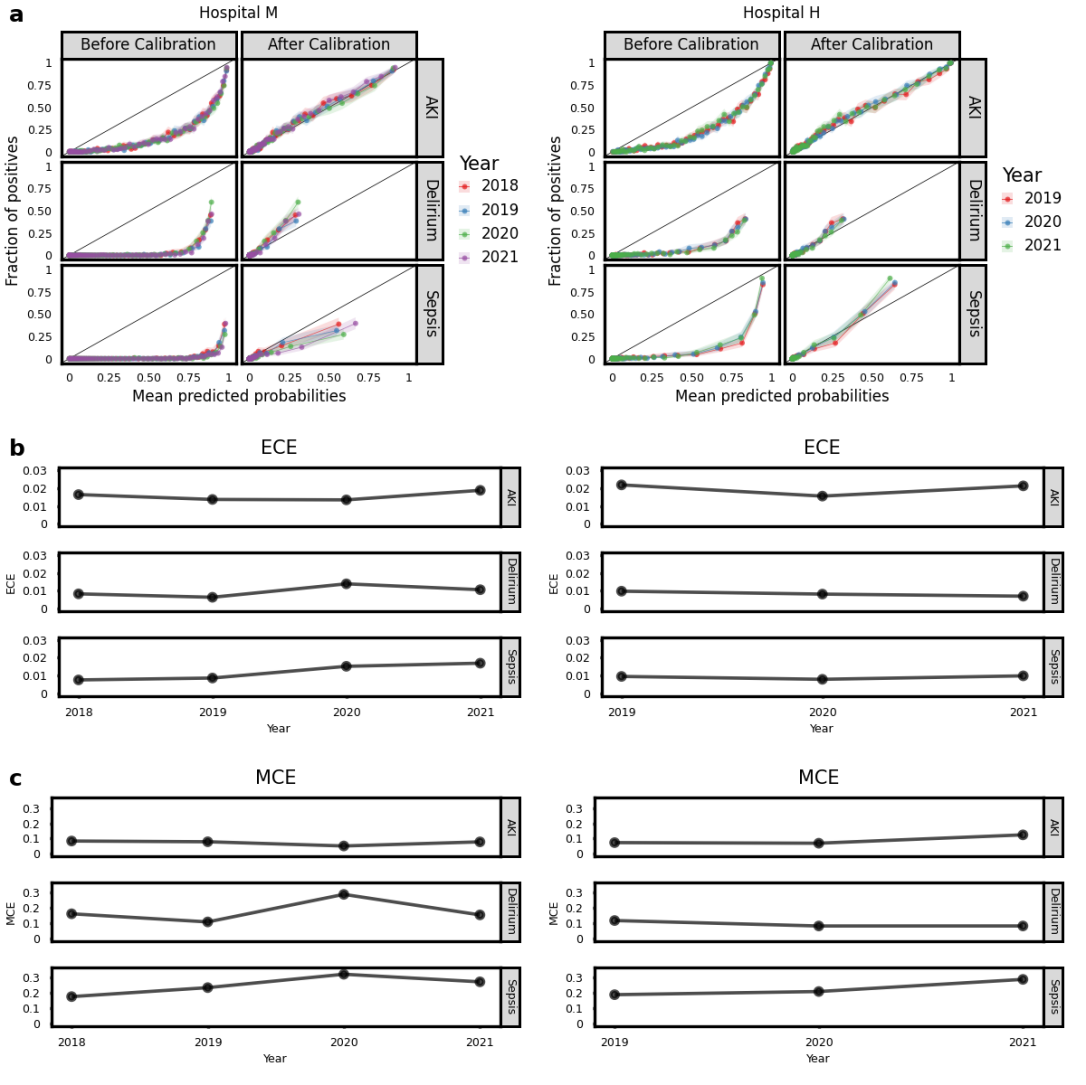
Calibration curves and corresponding ECE and MCE values: (a) calibration curves for each use case and year before and after calibration, with 95% CIs; (b) ECE values across the years for each use case; and (c) MCE values over the years for the 3 use cases. AKI: acute kidney injury; ECE: expected calibration error; MCE: maximum calibration error.

In addition to the evaluation of the AUROC, which reflects model performance, we also investigated whether there was any potential shift in model calibration. Figure 2 shows the calibration curves, as well as their corresponding ECE and MCE values. Figure 2a displays the calibration curves of the 2 use cases at hospitals M and H, respectively. The curves before calibration indicated that the averaged predicted risks were higher than the observed event rate, which means our models overestimated the risk. Consequently, we calibrated our models by applying the Platt scaling method [27]. The curves after calibration showed that the predicted risks better matched the observed positive ratio.

To better quantify the quality of the calibration curves, corresponding ECE (Figure 2b) and MCE (Figure 2c) values were generated for each calibrated curve. The error graphs did not show a clear increase in the shift across the evaluated years for any of the use cases at both hospitals, although small fluctuations were observed in the ECE and MCE curves in the delirium use case at hospital M. Furthermore, Figure 2a demonstrates that unlike the AKI use case, the sepsis and delirium use cases exhibited a calibration shift at hospital M, with the curves deviating from the diagonal line for higher probabilities. At hospital H, there was no evident calibration shift for both AKI and delirium use cases, although a slight shift was observed in its sepsis model.

Given the observed calibration shifts in sepsis and delirium, in contrast to the stability in AKI, we extended our investigations to examine whether the observed shift was intricately associated with inherent characteristics of the distinct conditions or whether it was influenced by other factors, such as variation in data size or incidence rates. Our results demonstrated that reducing the data size or incidence rates did not result in a calibration shift, and therefore, the observed calibration shift in sepsis and delirium could be use case specific or influenced by other factors (see Multimedia Appendix 2 for more details).

### Decision Evaluation

The last evaluation focused on decision-making analysis. We first assessed the alert rates, that is, the number of alerts triggered when the predicted probability exceeds the predefined threshold. We observed a slight increase over the years for all 3 use cases, even though the AUROC and the incidence remained similar at hospital M (see Table 2). However, we did not observe a similar increase at hospital H (see Table 3), where both incidence and alert rates remained stable.

**Figure 3:**
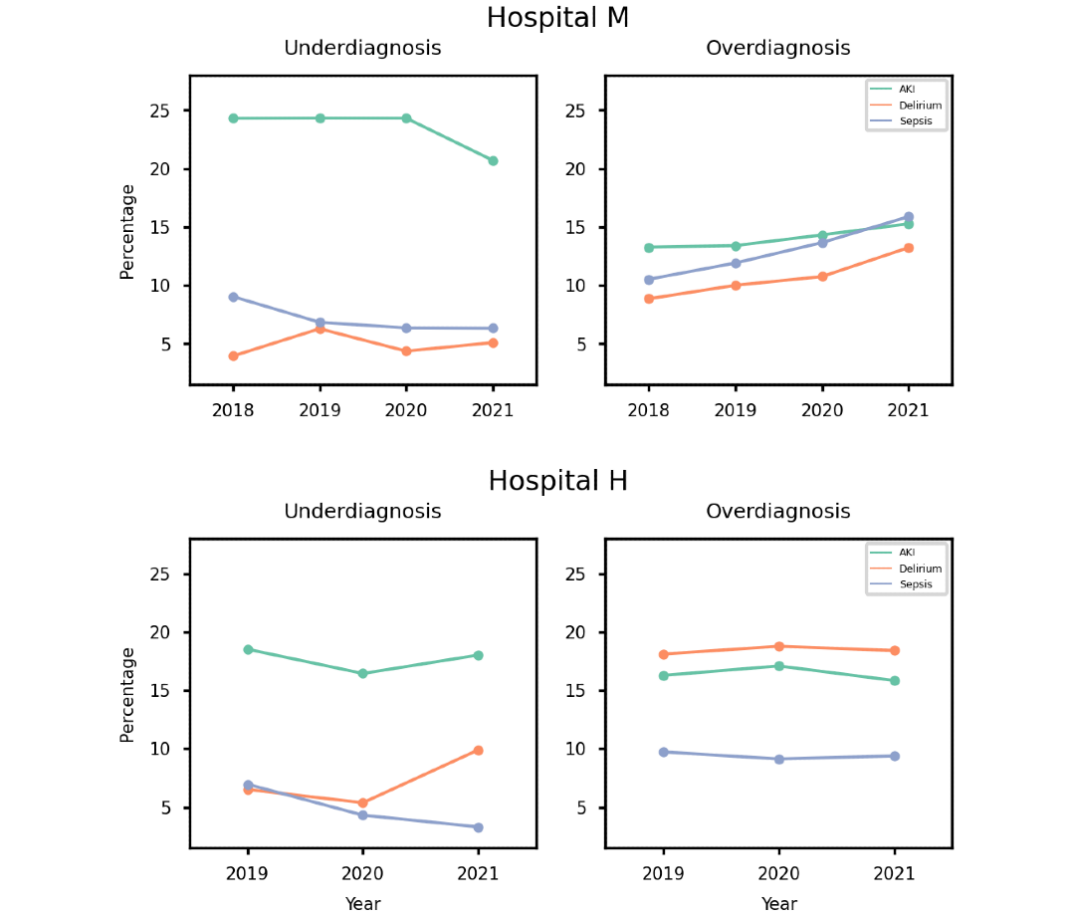
Under- and overdiagnosis rates across years in hospitals M and H. AKI: acute kidney injury.

The over- and underdiagnosis rates were further evaluated based on the correctness of an alert. The thresholds selected from the threshold selection set (10% of the training set) at hospital M were 19.97, 2.40, and 2.67 for AKI, delirium, and sepsis, respectively, and at hospital H were 23.13, 2.79, and 2.93, respectively. Figure 3 shows the under- and overdiagnosis rates for the test sets from the 3 use cases on a yearly basis at both hospital M and hospital H. At hospital M, we also observed that although the underdiagnosis rate remained stable over the years, there were clear trends of an increase in the overdiagnosis rate in all 3 use cases. Such an increase in overdiagnosis is in line with the increase in the alert rate and is likely associated with the increased number of observations in patient records (Tables S1 and S2 in Multimedia Appendix 1), as observed in the data analysis. The maximum deviation of the under- and overdiagnosis curves was around 5% in overdiagnosis on sepsis observed at hospital M, which aligns with the fact that the alert rate also increased. Such an increase is partially in line with the report that the alert rate of sepsis prediction models increased during the COVID-19 pandemic [19,20]. However, in this investigation, the trend of such an increase started before the pandemic.

At hospital H, there were more fluctuations in the underdiagnosis rate. The 3 use cases behaved differently, hindering generalization. Although the number of observations in patient records also increased yearly at hospital H, in contrast to hospital M, its overdiagnosis rates remained stable for all 3 use cases. Furthermore, the underdiagnosis rate of sepsis decreased during the COVID-19 pandemic, while its overdiagnosis rate remained stable. This indicates that the performance of the sepsis prediction model at hospital H improved during the COVID-19 pandemic, which contradicts the findings of hospital M, as well as other reports [19,20].

In summary, the 2 hospitals behave differently in terms of over- and underdiagnosis rates. Therefore, site-specific monitoring schemes are required to detect such changes. Consequently, given the difficulty in determining whether a model shift occurred based solely on the under- and overdiagnosis rates, we additionally performed DCA to test for any changes in the clinical utility of the models.

To examine the potential impact of the model on decision-making over time, DCA was performed for each distinct year and use case. The net benefit was calculated across the full range of probability thresholds. However, the lower range of the probability thresholds is more relevant to clinicians as, arguably, most clinicians would agree that the medical and financial consequences of delirium, sepsis, and AKI outweigh the nuisance to the user and the cost and inconvenience of preventive measures and closer monitoring. It is necessary to find the optimal threshold that has a good balance between FPs and FNs to prevent the excessive generation of unnecessary alerts, which can lead to user desensitization and decreased responsiveness. Additionally, it is important to highlight that the initial position of the decision curves (threshold=0) is notably affected by the incidence rate of the particular use case. Therefore, to ensure that the differences between the curves did not stem from different incidence rates, we standardized the incidence rate across the years by augmenting the incidence of the years that had lower rates until a uniform incidence was achieved across all years.

**Figure 4:**
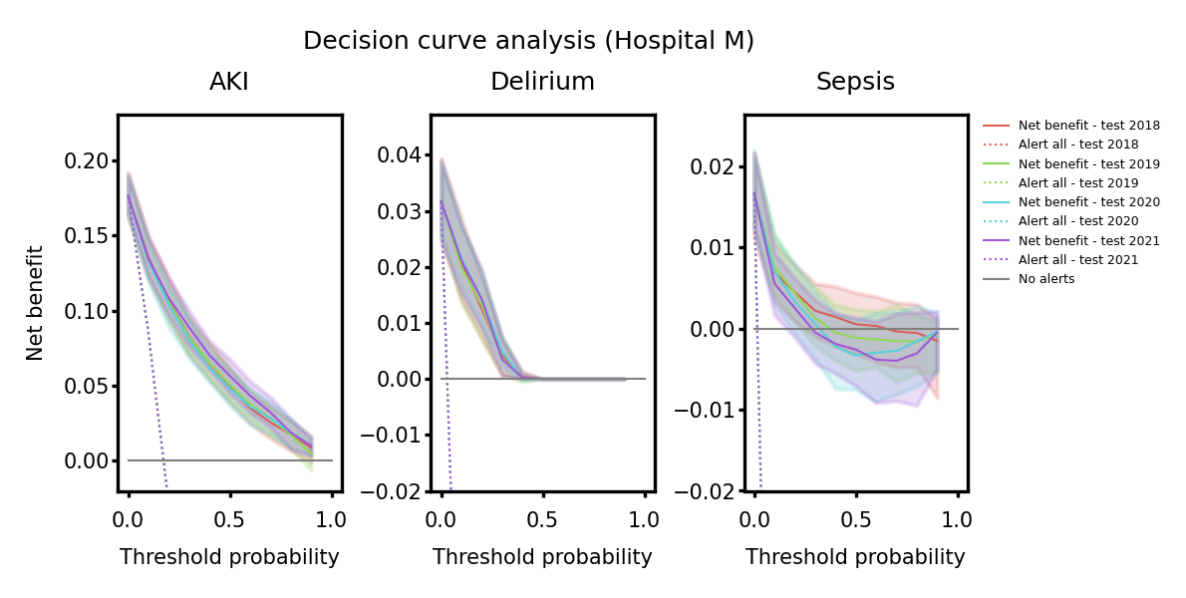
Decision curves for hospital M. Colored dotted lines show the net benefit of alerting all patients, gray lines represent the benefit of not alerting any patient, and colored solid lines show the net benefit of using the model on each of the years in the test. AKI: acute kidney injury.

Figure S1 in Multimedia Appendix 2 and Figure 4 show the decision curves for hospitals M and H, respectively, for each year across the 2 use cases. To test whether there was a significant difference in the decision-making between the years, we performed a pairwise nonparametric statistical comparison between the decision curves from 2 individual years for all possible year pairs [33,34]. For all the use cases and hospitals, we did not find any significant difference between any of the years (all *P*>.05) at the thresholds of interest. These results demonstrated that the model did not experience a degradation in clinical utility across the years.

## Discussion

### Principal Findings

This paper evaluated the impact of a model shift on ML-based clinical risk prediction models. There was no significant model shift in the period under this investigation. The models’ performances remained stable for all the 3 use cases, and there were no significant differences in the AUROCs between the years. Nevertheless, analysis of the calibration curves revealed a calibration shift in some use cases. The calibration curves of AKI were more stable compared to those for the delirium and sepsis prediction models at both hospitals in this study. No calibration shift was observed for AKI, even when the size of the sample and the rate of incidence were downscaled. These results suggest an intrinsic difference among the different use cases. Lastly, with respect to the interpretation of the predictions, the rate of alerts and overdiagnosis behaved differently across different hospitals: they consistently increased in the 3 use cases at hospital M and remained stable at hospital H. Furthermore, the examination of DCA results revealed a consistent pattern where the models exhibited no deterioration over time, affirming the stability of their clinical utility and decision-making.

These findings suggest that relying solely on AUROCs, the commonly used metric for assessing the performance of clinical risk prediction models, is inadequate in capturing the impact of a model shift. It is, therefore, essential to use comprehensive evaluations, such as calibration curve analysis, alert rate monitoring, and DCA, to promptly detect any kind of model shift. Additionally, recalibrating the model when the calibration curve significantly deviates from the diagonal may offer a viable solution without necessitating complete model retraining. This approach is effective because the chosen probability calibration approach does not affect the evaluation metrics as AUROCs or AUPRCs. However, in other situations where the performance or clinical usefulness of the model has deteriorated, retraining the model would be necessary. Furthermore, this study revealed that the potential impact of a model shift cannot be easily generalized, as the evaluations exhibited diverse behaviors across different use cases and hospitals. Therefore, implementing proper model calibration is crucial to avoid undesirable consequences, such as a significant increase in the alert rate [19,20].

### Comparison With Prior Works

Previous research that investigated model shifts in the clinical domain often worked with a single use case at a single site. For instance, Davis et al [14] examined data shift scenarios in AKI risk prediction with 1 data set. Similarly, Minne et al [15] used a single data set to evaluate the effect of temporal changes on mortality prediction performance. Therefore, later studies have argued that the focus on developing new models in the clinical domain should introduce more heterogeneity in model development and model evaluation [16]. The study presented in this paper leveraged 2 large data sets from different hospitals and investigated 3 distinct use cases, thereby enhancing the generalizability of the findings. Furthermore, although previous studies [14,15] have focused on analyzing model drift in terms of performance, this study extended the evaluation to a broader set of metrics, assessing the evolution of clinical utility of the model over time. This approach significantly enhances the heterogeneity and robustness of the evaluation.

### Motivations

ML-based prediction models are often closely tied to the system environment in which the models undergo training. After the models are deployed as real-world applications, their performance may deteriorate over time when the system experiences changes in its environment. The consequences of such changes largely impact the reliability of ML-based prediction models. Although the dynamic nature of the system is inevitable, timely detection of its impact is, therefore, of utmost importance to prevent model deterioration when it is used in clinical settings. This study aimed to investigate various approaches for monitoring the implications of model shifts by examining diverse use cases across different types of hospitals.

### Strengths

This study performed comprehensive evaluations of model behaviors over a 4-year period. The evaluation was not limited to model performance, also assessing the consequence of the model shift on clinical decision-making. Such detailed evaluations were performed on 3 different use cases at both a specialized hospital and a generalized hospital so as to better generalize the findings of our study. To the best of our knowledge, this study is the first report to investigate the consequences of a model shift on such a comprehensive level and scale.

### Limitations

This study has some limitations. First, the study was performed on retrospective data to simulate how prediction models would behave over time after they were trained. Evaluating model behavior in real-world clinical settings would provide more valuable insights. Second, the cause of the calibration shift observed on the delirium and sepsis use cases was not thoroughly analyzed in this study. We hypothesized that such a difference may be related to the different labeling strategies: the delirium and sepsis use cases were labeled with discharge ICD codes, while the AKI use case was labeled with the KDIGO algorithm. The use of ICD codes is imperfect owing to inevitable variations and inconsistencies in coding practice. However, lacking standard criteria for diagnosis, the use of such codes is an established method for retrospective identification [36]. Discharge ICD codes are likely more vulnerable to changes in coding policies, while diagnostic algorithms, such as KDIGO, that rely on lab values are less likely to change over time.

### Future Directions

This study evaluated the impact of a model shift on ML-based clinical risk prediction models. As a future step, we plan to conduct the discussed evaluations on our prediction models after they are deployed in live EHR systems. In addition, we will further investigate the different behaviors of calibration curves between the AKI use case and the other 2 use cases. Specifically, we aim to explore the influence of various labeling policies on these behaviors.

Including retrospective data from earlier years may help build a more reliable model with more training samples; however, it may also increase the risk of data shift. We will further investigate how to make a good trade-off when selecting training data, thus significantly influencing the development of a good prediction model, while minimizing the model shift.

### Conclusion

This study performed evaluations across different hospitals to investigate the impact of model shifts on various ML-based risk prediction models. Although the metrics of AUROCs remained stable for the evaluated use cases over the years, the analysis of calibration curves varied across certain use cases (ie, delirium and sepsis) at different hospitals. However, upon conducting more in-depth investigations using DCA, it was confirmed that these variations did not have any impact on the clinical utility of the models, as there was no evident degradation in their decision-making capabilities. Moreover, the COVID-19 pandemic has the potential to alter clinical diagnosis and treatment patterns over the long term. So, to obtain a more complete understanding of this impact in prediction models, a future study incorporating additional years of data would be highly valuable. As a summary, these findings suggest the need for appropriate monitoring schemes and timely model calibrations following the deployment of prediction models.

## Appendix

Multimedia Appendix 1 Data characteristics in hospitals H and M for each year, number of features used in hospitals H and M, leaking features, and *Z* test results for hospitals H and M.

Multimedia Appendix 2 Decision and calibration curve analysis results.

## Abbreviations

AKI: acute kidney injury
AUPRC: area under the precision recall curve
AUROC: area under the receiver operating characteristic curve
DCA: decision curve analysis
ECE: expected calibration error
EHR: electronic health record
FN: false negative
FNR: false-negative rate
FP: false positive
FPR: false-positive rate
ICD-10-GM: German Modification of the International Statistical Classification of Diseases and Related Health Problems, Tenth Revision
KDIGO: Kidney Disease Improving Global Outcomes
MCE: maximum calibration error
ML: machine learning
NEN: named entity normalization
NER: named entity recognition
NLP: natural language processing
TN: true negative
TP: true positive

## Data Availability

Due to commercial restrictions, legal obligations, and data protection, data/code availability is not applicable to this paper.
