# Supplementary material for: Longitudinal Model Shifts of Machine Learning–Based Clinical Risk Prediction Models: Evaluation Study of Multiple Use Cases Across Different Hospitals
Source: J Med Internet Res. 2024 Dec 13;26:e51409. doi: 10.2196/51409 (PMC11681292; doi:10.2196/51409)
Supplement: Multimedia Appendix 1 [file jmir_v26i1e51409_app1.docx]

**Table S1.** Data characteristics in hospital M for each year.

| Hospital M | Medical cases | Incidence (%) | | | Average length of observation (number of features) | | | Average length of Stay (days) | Gender distribution  (- men, + women %) | Memos | Medi-cation | Lab results | Vital signs |
| --- | --- | --- | --- | --- | --- | --- | --- | --- | --- | --- | --- | --- | --- |
|  |  | AKI | DEL | SEP | AKI | DEL | SEP |  |  |  |  |  |  |
|  |  |  |  |  |  |  |  |  |  |  |  |  |  |
| **2009** | 12544 | 16.73 | 0.40 | 0.87 | 18.81 | 19.79 | 22.45 | 6.6 | 6.55 | 72500 | 1 | 716561 | 0 |
| **2010** | 12110 | 16.16 | 0.26 | 1.03 | 20.57 | 21.94 | 25.42 | 6.3 | 5.88 | 81588 | 0 | 714994 | 0 |
| **2011** | 12599 | 14.52 | 1.79 | 1.13 | 21.64 | 24.97 | 27.33 | 6.0 | 4.10 | 84134 | 0 | 714474 | 0 |
| **2012** | 12793 | 15.86 | 1.71 | 0.90 | 25.22 | 29.57 | 30.16 | 5.9 | 5.17 | 115006 | 0 | 739024 | 0 |
| **2013** | 13851 | 16.14 | 1.75 | 1.17 | 29.78 | 33.00 | 36.95 | 5.5 | 1.12 | 168395 | 1 | 900678 | 195 |
| **2014** | 14775 | 15.05 | 2.39 | 1.34 | 32.08 | 39.52 | 39.71 | 5.2 | 0.89 | 226237 | 0 | 995955 | 320 |
| **2015** | 14929 | 16.15 | 2.47 | 1.98 | 33.82 | 40.36 | 44.12 | 5.2 | 1.35 | 315498 | 11 | 1031251 | 3758 |
| **2016** | 16237 | 16.14 | 1.84 | 1.74 | 42.99 | 53.63 | 61.98 | 5.0 | 2.15 | 572628 | 18159 | 1342602 | 296680 |
| **2017** | 16403 | 16.11 | 2.17 | 2.21 | 60.98 | 80.05 | 85.43 | 5.0 | 1.18 | 1358212 | 22334 | 1417821 | 732124 |
| **2018** | 16300 | 16.63 | 3.06 | 2.10 | 62.57 | 62.57 | 62.57 | 5.2 | 3.52 | 1867320 | 22475 | 1417685 | 1036786 |
| **2019** | 17045 | 16.14 | 2.61 | 1.60 | 66.38 | 66.38 | 66.38 | 5.1 | 3.00 | 2013480 | 22757 | 1453571 | 1107172 |
| **2020** | 15263 | 16.52 | 3.90 | 1.38 | 70.41 | 70.41 | 70.41 | 5.1 | 5.09 | 1852411 | 20694 | 1483798 | 1017104 |
| **2021** | 14926 | 19.18 | 3.41 | 1.90 | 73.81 | 73.81 | 73.81 | 5.3 | 5.53 | 1755647 | 21704 | 1694921 | 1051909 |

**Table S2.** Data characteristics in hospital H for each year.

| Hospital H | Medical cases | Incidence (%) | | | Average length of observation (number of features) | | | Average length of Stay (days) | Gender distribution  (- men, + women %) | Memos | Medi-cation | Lab results | Vital signs |
| --- | --- | --- | --- | --- | --- | --- | --- | --- | --- | --- | --- | --- | --- |
|  |  | AKI | DEL | SEP | AKI | DEL | SEP |  |  |  |  |  |  |
|  |  |  |  |  |  |  |  |  |  |  |  |  |  |
| **2017** | 14157 | 23.88 | 2.08 | 2.35 | 77.12 | 82.72 | 82.59 | 9.5 | -34.24 | 1687235 | 0 | 3184884 | 753258 |
| **2018** | 13298 | 24.03 | 3.30 | 3.41 | 87.55 | 95.11 | 95.24 | 9.4 | -33.27 | 1340519 | 158424 | 3083755 | 691710 |
| **2019** | 14601 | 23.65 | 2.78 | 3.55 | 92.26 | 99.33 | 99.38 | 13.9 | -34.53 | 1097365 | 313513 | 3171839 | 729609 |
| **2020** | 13211 | 25.20 | 2.90 | 3.66 | 95.99 | 104.17 | 104.50 | 14.9 | -33.36 | 1012060 | 280960 | 3085791 | 680117 |
| **2021** | 13742 | 25.04 | 2.61 | 3.73 | 98.98 | 109.00 | 108.08 | 14.6 | -33.26 | 1115709 | 301449 | 3059734 | 741349 |

**Table S3.** Number of features used in hospitals M and H.

| Feature group - Feature value | | Hospital M | Hospital H |
| --- | --- | --- | --- |
| **Gender** | |  |  |
|  | Male | 2 | 2 |
|  | Female |  |  |
| **Age group** | |  |  |
|  | 1 [18, 25] | 9 | 9 |
|  | 2 [26, 35] |  |  |
|  | 3 [36, 45] |  |  |
|  | 4 [46, 55] |  |  |
|  | 5 [56, 65] |  |  |
|  | 6 [66, 75] |  |  |
|  | 7 [76, 85] |  |  |
|  | 8 [86, 95] |  |  |
|  | 9 ≥ 95 |  |  |
| **ICD historical** | | 4499 | 1697 |
| **Memo ICD** | | 5277 | 4009 |
| **Medication** | | 336 | 1609 |
| **Lab results** | |  |  |
|  | LL (very low) | - | 15 |
|  | L (low) | 89 | 101 |
|  | N (normal) | - | - |
|  | H (high) | 132 | 160 |
|  | HH (very high) | - | 36 |
| **Vital sign** | | 30 | 30 |
| Total |  | 10386 | 7668 |

**Table S4.** Leaking features.

| Feature type | AKI | Delirium | Sepsis |
| --- | --- | --- | --- |
|  |  |  |  |
| **Memo_ICD** | N14.*, N17.*, N19, N99.0, R34, R94.4 | F05, F1*.4 | A02.1, A20.7, A21.7, A22.7, A24.1, A26.7, A28.0, A32.7, A33, A39.1, A39.2, A39.3, A39.4, A40, A41, A42.7, A48.0, A48.3, B00.7, B37.7, B44.7, I33.0, M86.0, O85, O88.3, P36, R57.2, R65 |
| **Lab results** | HARNSTOFF, KREA*, CREA*, HARNSAEURE, KRISTALLE, PATH*ZYLINDER, HEFEZELLEN, RUNDEPITHELIEN, MICROALB, GLOM | None | IL_6, PROCALC* |
| **Medication** | FUROSEMID | None | None |

**Table S5.** Z-test results for hospital M.

| Hospital M | Years | Z score | p-value |
| --- | --- | --- | --- |
| AKI | |  |  |
|  | 2018-2019 | 0.181 | 0.857 |
|  | 2018-2020 | 0.895 | 0.370 |
|  | 2018-2021 | -0.202 | 0.839 |
|  | 2019-2020 | 0.746 | 0.455 |
|  | 2019-2021 | -0406 | 0.684 |
|  | 2020-2021 | -1.171 | 0.242 |
| Delirium | |  |  |
|  | 2018-2019 | 0.603 | 0.546 |
|  | 2018-2020 | 0.344 | 0.731 |
|  | 2018-2021 | 1.235 | 0.216 |
|  | 2019-2020 | -0.323 | 0.747 |
|  | 2019-2021 | 0.584 | 0.559 |
|  | 2020-2021 | 0.995 | 0.319 |
| Sepsis | |  |  |
|  | 2018-2019 | -0.049 | 0.960 |
|  | 2018-2020 | 0.502 | 0.615 |
|  | 2018-2021 | 0.487 | 0.625 |
|  | 2019-2020 | 0.553 | 0.580 |
|  | 2019-2021 | 0.543 | 0.586 |
|  | 2020-2021 | -0.058 | 0.953 |

**Table S6.** Z-test results for hospital H.

| Hospital H | Years | Z score | p-value |
| --- | --- | --- | --- |
| AKI | |  |  |
|  | 2019-2020 | 1.265 | 0.205 |
|  | 2019-2021 | -1.072 | 0.283 |
|  | 2020-2021 | 0.212 | 0.831 |
| Delirium | |  |  |
|  | 2019-2020 | -0.263 | 0.792 |
|  | 2019-2021 | 0.846 | 0.397 |
|  | 2020-2021 | 0.587 | 0.556 |
| Sepsis | |  |  |
|  | 2019-2020 | 1.021 | 0.307 |
|  | 2019-2021 | -1.395 | 0.163 |
|  | 2020-2021 | -0.368 | 0.712 |
