# Supplementary material for: Longitudinal Model Shifts of Machine Learning–Based Clinical Risk Prediction Models: Evaluation Study of Multiple Use Cases Across Different Hospitals
Source: J Med Internet Res. 2024 Dec 13;26:e51409. doi: 10.2196/51409 (PMC11681292; doi:10.2196/51409)
Supplement: Multimedia Appendix 2 [file jmir_v26i1e51409_app2.docx]

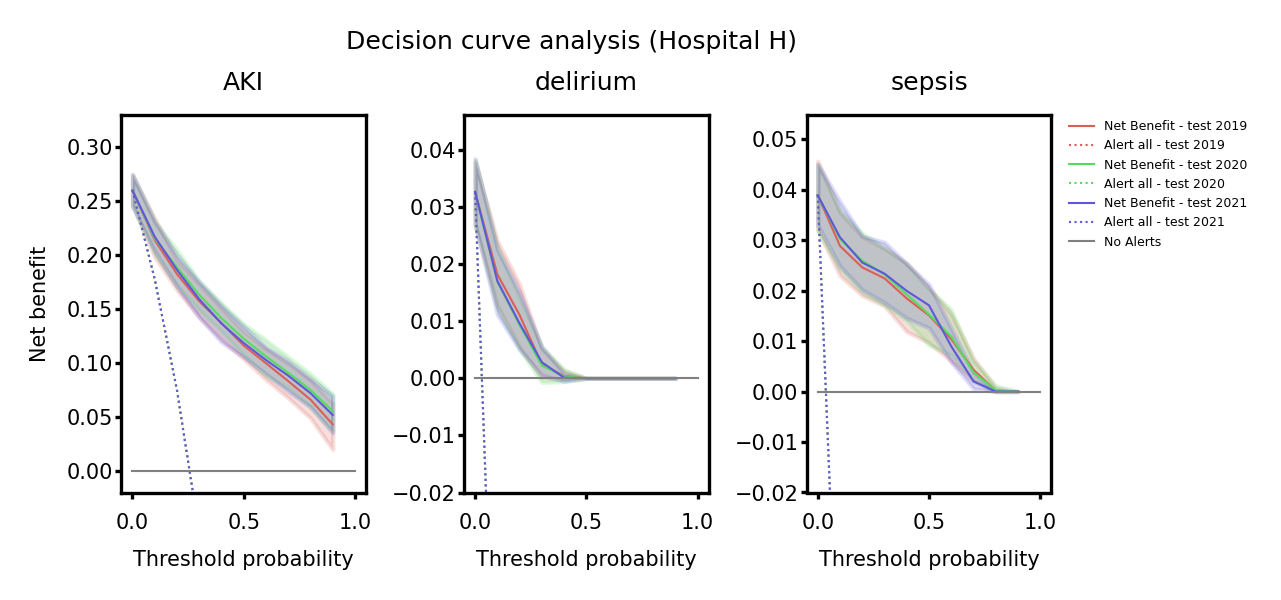


**Figure S1.** Decision curves for hospital H. Colored dotted lines show the net benefit of alerting all the patients, gray lines represent the benefit of not alerting any patient, and colored solid lines show the net benefit of using the model on each of the years in the test. AKI: acute kidney injury.

### NLP Models

Named entity recognition (NER) model: The NER model was developed using BERT (Bidirectional Encoder Representations from Transformers). To train the BERT model, we used a pretrained German BERT model that was trained on unlabeled data from a large-volume corpus. Then, the BERT model was fine-tuned on an NER task using data from 6 hospitals to detect clinical entities (disorders and findings). To improve the speed of the NER task, a TinyBERT NER model was further distilled from the BERT NER model.

Named entity normalization (NEN) model: We used an approach that normalizes clinical terms to ICD-10-GM codes by taking into account semantic and syntactic information [1]. The normalization model was trained to map the extracted NER mentions from 6 hospitals and their synonyms to their appropriate ICD codes.

The NER and NEN models were evaluated on in-house evaluation sets that were manually labeled by clinical professionals. The model performance was evaluated by comparing the predicted NER entities and their ICD labels with human-defined labels.

For the data preparation in our main experiment, the NER pipeline consisted of using the NER model to extract clinical entities from the clinical notes. The extracted entities were mapped to their corresponding ICD codes using the normalization model. The normalized features were then used as an input to our risk prediction models. The performance of the NER model was 0.78, 0.83, and 0.82 for the *F*_1_-score, precision, and recall, respectively.

### Downscaling Investigations

The main difference between the AKI and delirium and sepsis use cases was that the former had a larger number of samples and a higher incidence compared to the latter 2 (Table 1). This was due to the clinically-based KDIGO labeling approach that was used for AKI [2] (as opposed to the billing-oriented ICD code approach used in the other use cases). Therefore, we downscaled the AKI samples to investigate whether the absence of a calibration shift in AKI could be attributed to the large data set size or high incidence. We first reduced the samples of the AKI data sets, not only for training but also for calibration and testing, without changing the incidence rate. Features for the remaining sample were not removed and were kept intact. Details of the data set information are provided in Table S1 in Multimedia Appendix 1. Figures S2 and S3 in Multimedia Appendix 2 show the results of this experiment. The downscaling to 11% approximated the ratio of the size of the delirium or sepsis data sets compared to the AKI data set. The graphs presented in Figures S2 and S3 in Multimedia Appendix 2 demonstrated that neither reducing the data set size for AKI nor diminishing the training set size noticeably impacted the calibration curves. However, when the size of the calibration/test set was reduced, increased fluctuations were observed within the calibration curves. This effect was due to a decrease in the number of predictions within each bin, leading to a larger sampling error and introducing noise to the curve.


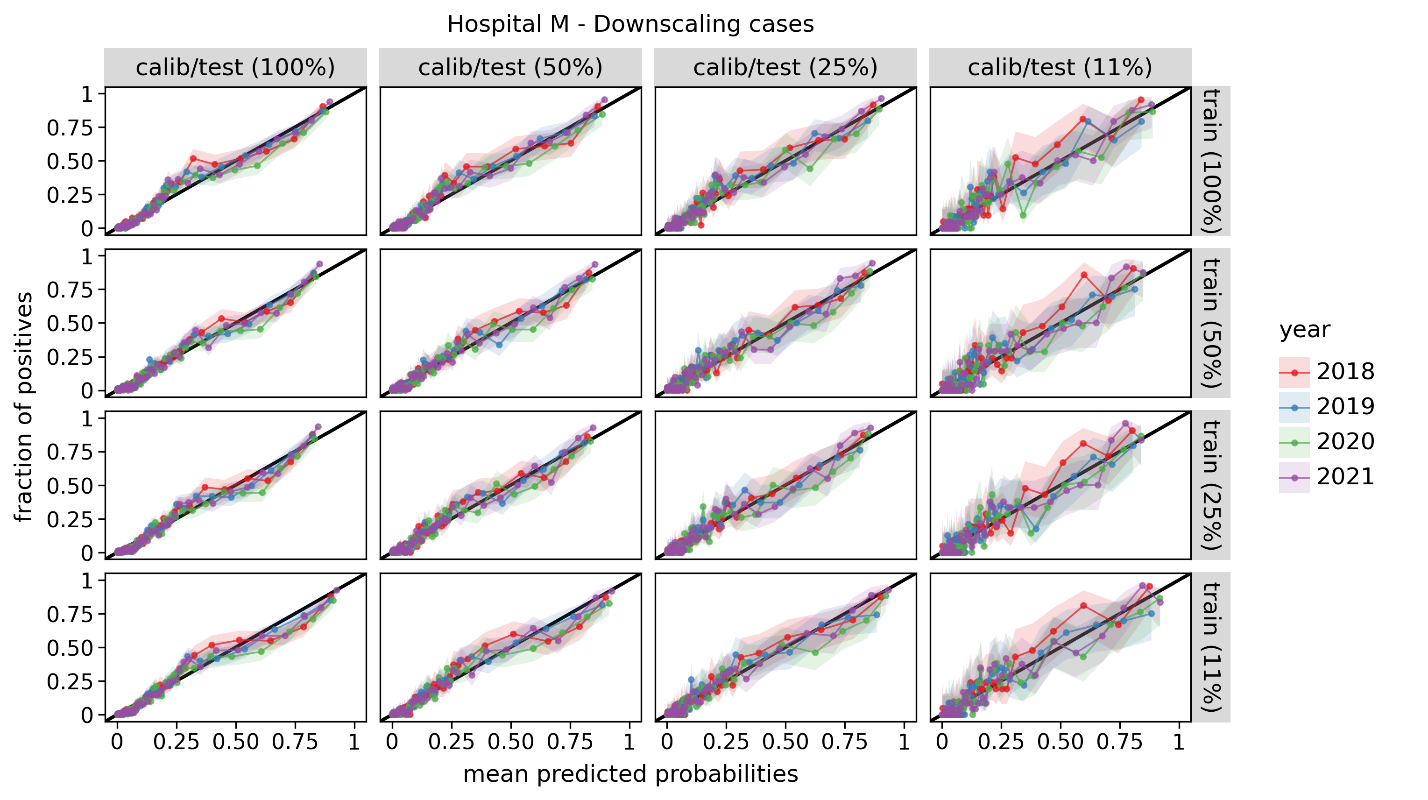


**Figure S2.** Calibration curve results when downscaling the data set size in AKI data sets for hospital M without changing the incidence. The percentages after the training or calibration/test sets indicate the rate of remaining samples compared to the original set. AKI: acute kidney injury.


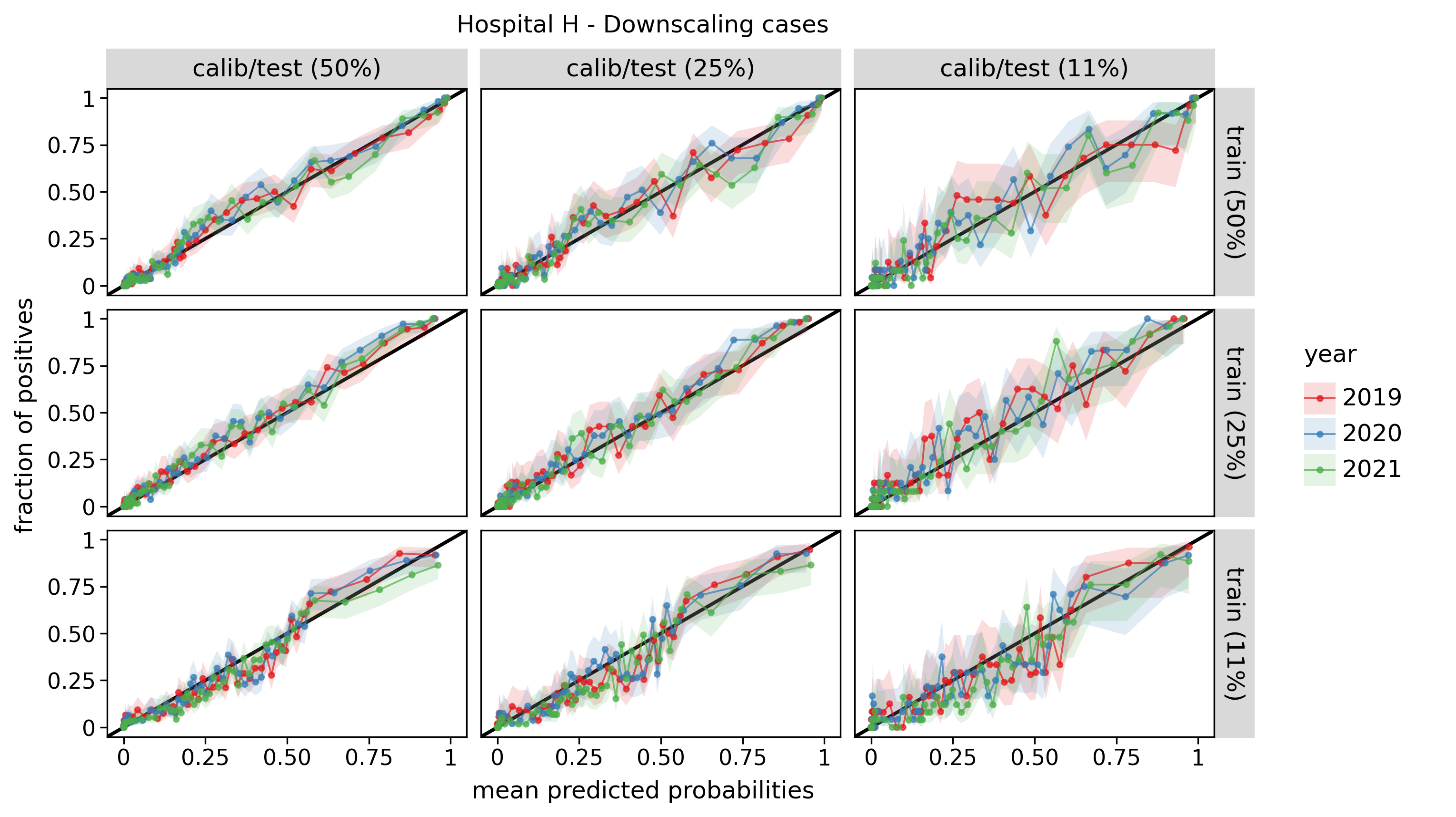


**Figure S3.** Calibration curve results when downscaling the data set size in AKI data sets for hospital H without changing the incidence. The percentages after the training or calibration/test sets indicate the rate of remaining samples compared to the original set. AKI: acute kidney injury.

We also investigated reducing the incidence of AKI to match the delirium incidence by downscaling the case: control ratio in the calibration and test sets. For the training set, the rate between positive and negative cases were fixed to 1:5 following our default training policy [3]. This allowed us to evaluate the impact of reduced incidence on different scales of data. Figures S4 and S5 show the result of this experiment. The downscaling to 11% approximated the ratio of the incidence of the delirium or sepsis use case compared to the AKI use case.

The results depicted in Figures S4 and S5 provide compelling evidence that reducing the incidence had no apparent impact on calibration shift, and when decreasing the calibration and test case: control ratio, this merely increased the variability/variance of the calibration curve. The observed results demonstrate congruence across both hospitals, indicating consistency in the outcomes obtained from these experiments.


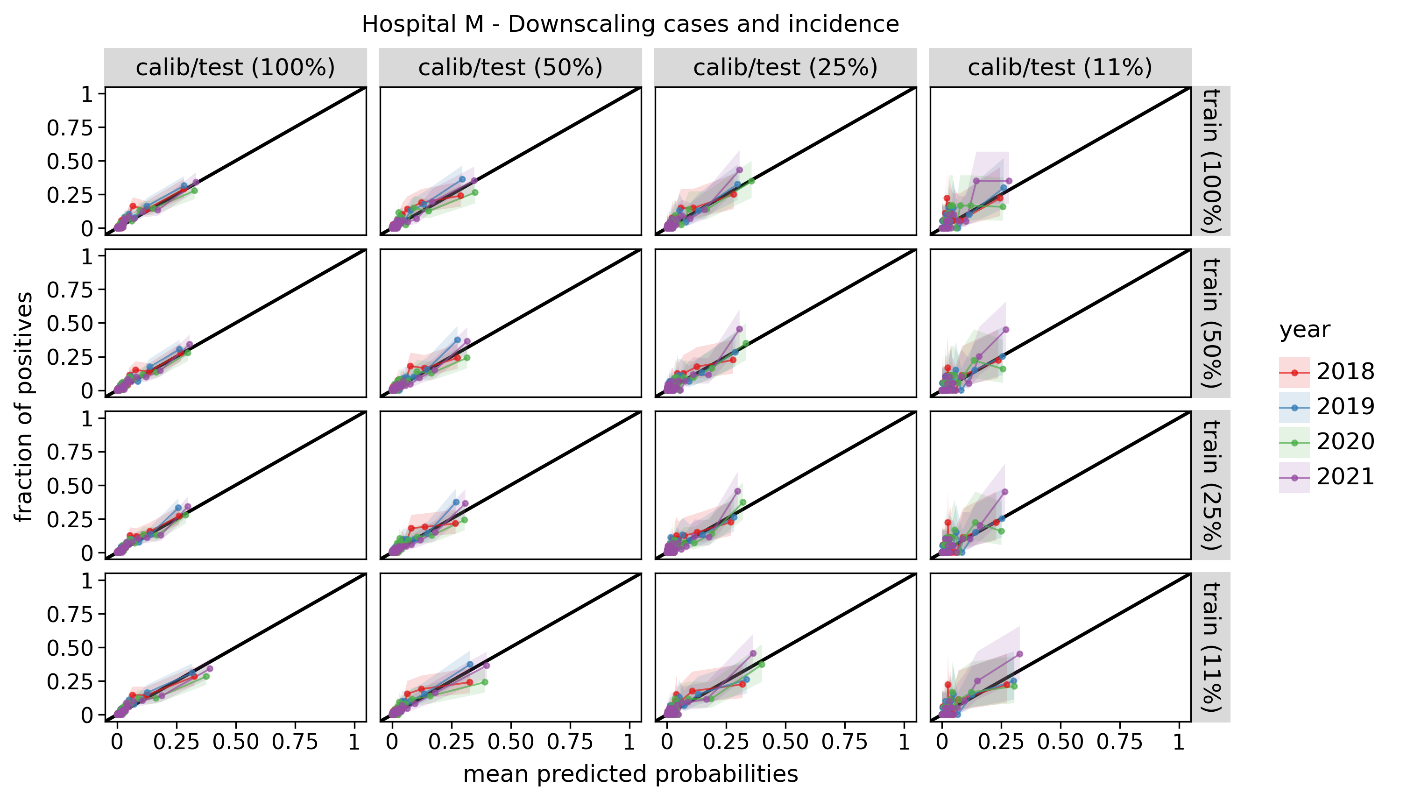


**Figure S4.** Calibration curves when reducing the data set size and the incidence to match that of delirium for hospital M. The percentages after the training or calibration/test sets indicate the relative case: control ratio in the downscaled data set compared to the original set.


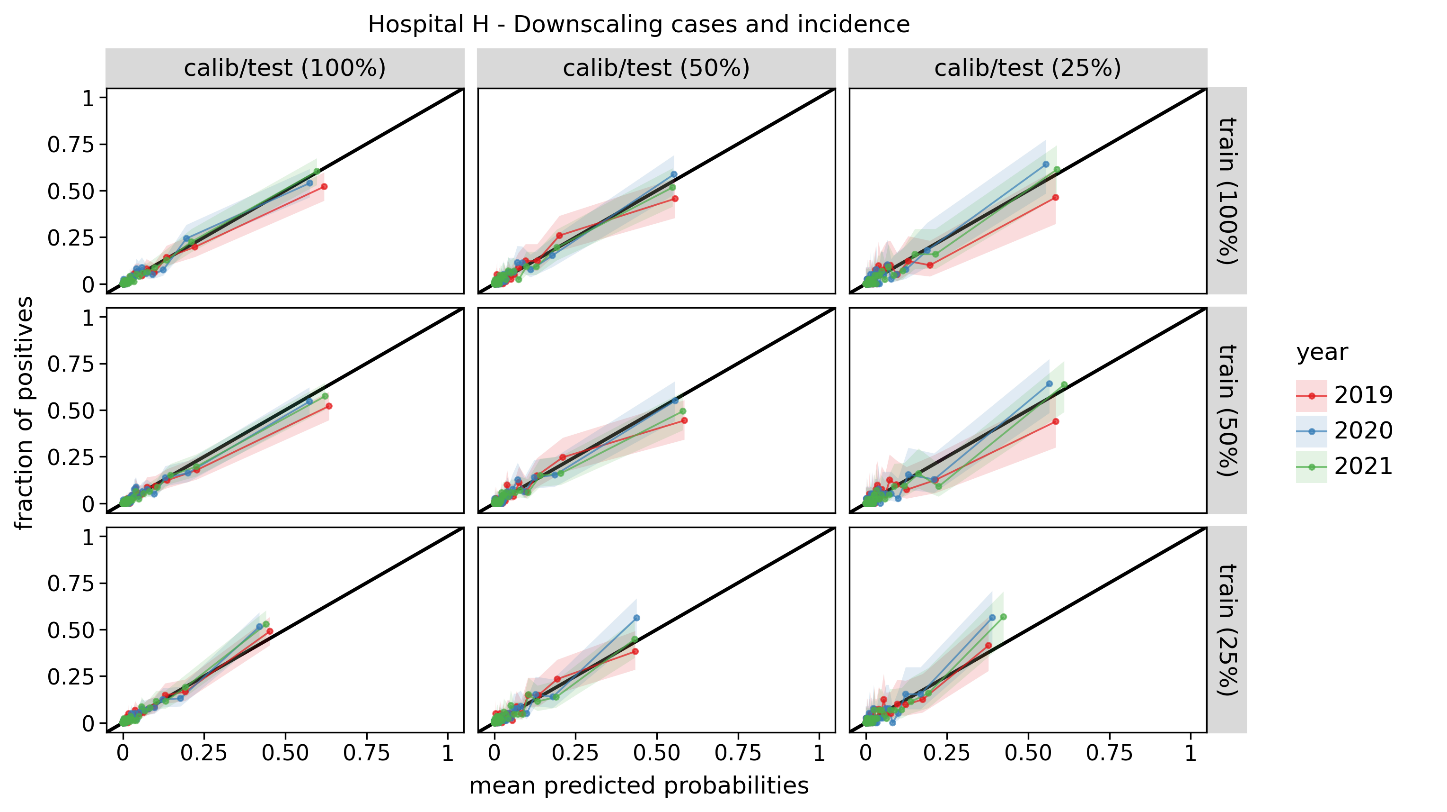


**Figure S5.** Calibration curves when reducing the data set size and the incidence to match that of delirium for hospital H. The percentages after the training or calibration/test sets indicate the relative case: control ratio in the downscaled data set compared to the original set.

## References

1. Sung M, Jeon H, Lee J, Kang J. Biomedical entity representations with synonym marginalization. arXiv:200500239 [cs] preprint posted online 2020. [doi:
   10.48550/arXiv.2005.00239]. 2020 May 1.
2. Khwaja A. KDIGO clinical practice guidelines for acute kidney injury. *Nephron Clin Pract* 2012;120(4):c179–c184.
3. Sun H, Depraetere K, Meesseman L, De Roo J, Vanbiervliet M, De Baerdemaeker J, Muys H, von Dossow V, Hulde N, Szymanowsky R. A scalable approach for developing clinical risk prediction applications in different hospitals. *J Biomed Inform* 2021 Jun; 118:103783.
